# Dating the Bacterial Tree of Life Based on Ancient Symbiosis

**DOI:** 10.1093/sysbio/syae071

**Published:** 2025-01-23

**Authors:** Sishuo Wang, Haiwei Luo

**Affiliations:** Simon F. S. Li Marine Science Laboratory, School of Life Sciences and State Key Laboratory of Agrobiotechnology, The Chinese University of Hong Kong, Tai Po Road, Shatin, Hong Kong SAR; Department of Microbiology, The Chinese University of Hong Kong, Tai Po Road, Shatin, Hong Kong SAR; Simon F. S. Li Marine Science Laboratory, School of Life Sciences and State Key Laboratory of Agrobiotechnology, The Chinese University of Hong Kong, Tai Po Road, Shatin, Hong Kong SAR; Institute of Environment, Energy and Sustainability, The Chinese University of Hong Kong, Tai Po Road, Shatin, Hong Kong SAR

**Keywords:** Molecular clock, bacterial tree of life, ancestral reconstruction of lifestyles, timetree, symbiosis, relative time constraint

## Abstract

Obtaining a timescale for bacterial evolution is crucial to understand early life evolution but is difficult owing to the scarcity of bacterial fossils. Here, we introduce multiple new time constraints to calibrate bacterial evolution based on ancient symbiosis. This idea is implemented using a bacterial tree constructed with genes found in the mitochondrial lineages phylogenetically embedded within Proteobacteria. The expanded mitochondria-bacterial tree allows the node age constraints of eukaryotes established by their abundant fossils to be propagated to ancient co-evolving bacterial symbionts and across the bacterial tree of life. Importantly, we formulate a new probabilistic framework that considers uncertainty in inference of the ancestral lifestyle of modern symbionts to apply 19 relative time constraints each informed by host-symbiont association to constrain bacterial symbionts no older than their eukaryotic host. Moreover, we develop an approach to incorporating substitution mixture models that better accommodate substitutional saturation and compositional heterogeneity for dating deep phylogenies. Our analysis estimates that the last bacterial common ancestor occurred approximately 4.0–3.5 billion years ago (Ga), followed by rapid divergence of major bacterial clades. It is generally robust to alternative root ages, root positions, tree topologies, fossil ages, ancestral lifestyle reconstruction, gene sets, among other factors. The obtained timetree serves as a foundation for testing hypotheses regarding bacterial diversification and its correlation with geobiological events across different timescales.

Bacteria are the dominating form of life in the first two-thirds of the history of our planet, and their evolution and diversification have greatly shaped the geosphere of Earth (Falkowski et al. 2008). Many hypotheses have been proposed to interpret outstanding questions like how bacteria affected the evolution of biogeochemical cycles or when major innovations of bacteria such as photosynthesis occurred (Battistuzzi et al. 2004; Battistuzzi and Hedges 2009; Louca et al. 2018). Interpreting these questions depends on temporal links between bacterial evolution and the corresponding geobiological events. However, efforts to date the early evolution of bacteria based on relaxed molecular clocks (Zuckerkandl and Pauling 1965; Drummond et al. 2006; Yang and Rannala 2006) often come to different conclusions. A major challenge is that biomarkers or fossils attributable to bacteria are scarce, and thus the nodes whose age can be constrained for bacterial timetree inference are few (Battistuzzi and Hedges 2009). In addition, because fossils or biomarkers by themselves provide only a lower minimum bound (Benton and Donoghue 2007; Marshall 2019), there are no reliable maximum time constraints to calibrate bacterial evolution. Accordingly, the time estimates heavily depend on the maximum age assigned to the root, that is, the last bacterial common ancestor (LBCA), whose origin time involves even more debate (Wang and Luo 2021; Moody et al. 2022). Moreover, previous studies in dating the early evolution of bacteria (Battistuzzi et al. 2004; Battistuzzi and Hedges 2009; Wang and Luo 2021) are mostly based on site-homogeneous amino acid substitution models that do not account for among-site compositional heterogeneity or substitutional saturation (Gouy et al. 2015). In deep-time phylogenetics, this may lead to an underestimate of branch length (expected number of substitutions per site) (Moody et al. 2022), which potentially affects time estimation.

Recent studies have used eukaryotic fossils to date the evolution of Proteobacteria (Wang and Luo 2021), ammonia-oxidizing bacteria and anaerobic ammonium-oxidizing bacteria (Liao et al. 2022, 2024), and Cyanobacteria (Sánchez-Baracaldo et al. 2017; Shih et al. 2017; Zhang et al. 2023) where mitochondrial (mito-) and/or plastid-endosymbiosis are involved. These successes imply the value of the abundant eukaryotic fossils in timing bacterial evolution using molecular clock-dating approaches. Here, we exploit a novel type of age constraint derived from bacterial endosymbiosis, which often bears a signature of a co-evolutionary history between symbiotic bacteria and their eukaryotic hosts. Unlike mitochondrial and plastid-endosymbiosis which are phylogenetically restricted to Proteobacteria and Cyanobacteria, respectively, bacterial endosymbiosis independently occurred in multiple ancient bacterial lineages (Toft and Andersson 2010; Perreau and Moran 2022), making it possible to propagate this novel timing information to the entire bacterial tree of life.

A recently developed framework called relative time constraint (RTC) dating (Davín et al. 2018; Magnabosco et al. 2018; Szöllõsi et al. 2022) seeks to improve divergence time estimation by constraining the order of divergence by assuming the age of some nodes of the tree to be younger than others. In previous works (Davín et al. 2018; Magnabosco et al. 2018; Szöllõsi et al. 2022), the order of divergence is often provided by horizontal gene transfer (HGT) where the recipient must be no older than the donor. In the present study and in the context of ancient bacterial symbiosis, RTCs may be based on the assumption that bacterial symbionts are no older than their eukaryotic hosts instead. Despite some encouraging progress, the previous implementation of this method treats all RTCs as if they are known with full certainty. However, this assumption might be oversimplified because of the uncertainty in identifying the relative time order based on HGT (Ravenhall et al. 2015) or other evolutionary events.

Here, we address the above challenges by leveraging combined evidence of molecular sequences, fossils, and ancient symbiosis to calibrate an integrative timescale of bacterial evolution. We developed and implemented a framework to propagate node age constraints of eukaryotes established by abundant fossils to ancient co-evolving bacterial symbionts and other bacteria by requiring that the origin of symbionts postdates their hosts’ while accommodating the uncertainty in ancestral host reconstruction. We established timelines of bacterial evolution at different taxonomy resolutions and discussed how they may affect our views of the evolution of early life and habitat.

## METHODS

### Phylogenomic Reconstruction

A total of 62 gene markers considered conserved across bacteria were retrieved from a recent phylogenomics study of the bacterial tree (Coleman et al. 2021). Their sequences were aligned using MAFFT v7.22261 (Katoh and Standley 2013) and trimmed using TrimAl v1.4 (settings: “-resoverlap 0.55 -seqoverlap 60”) (Capella-Gutiérrez et al. 2009). Taxonomy sampling is detailed in Supplementary Table S1 and Supplementary Note S1 (all Supplementary Material can be found in the Dryad data repository: https://doi.org/10.5061/dryad.1c59zw42s). Unrooted phylogenomic trees were constructed based on the concatenated alignment with time-reversible Markov model and each of the 3 empirical site-heterogeneous profile mixture models C20, C40, and C60 using a PMSF (posterior mean site frequency)  approximation (guide tree built by LG4M + G + I) with 1000 ultrafast bootstraps (Minh et al. 2020). The model LG + G + C60 was selected as the best-fit model according to AIC (Akaike information criterion) and BIC (Bayesian information criterion), and the tree constructed by this model was used in the main analysis. Rooted bacterial trees were constructed using the recently developed time-nonreversible model implemented in IQ-Tree v2.1.3 (Naser-Khdour et al. 2022) that took the unrooted tree as either a fixed tree or a starting tree (see Supplementary Table S2 and Supplementary Note S2 for details).

### Identification of Mitochondrial Genes Conserved Across the Bacterial Tree

We retrieved the sequences of the 108 genes identified to have a mitochondrial origin from the study carried out by Muñoz-Gómez et al. (2022). We identified their homologs in eukaryotes encoded by either the mitochondrial or nuclear genome based on BLAST v2.6.0 + with an e-value cutoff of 1e-30. Next, we followed Liao et al. (2022) to keep 32 genes present in at least one of the following 3 gene sets namely bac120 (120 genes present in nearly all bacteria that commonly serve as phylogenetic markers for distinguishing between bacterial lineages) (Parks et al. 2017), Battistuzzi2009 (25 genes) (Battistuzzi and Hedges 2009), and Coleman2021 (62 genes) (Coleman et al. 2021), which include orthologs well conserved and likely vertically transmitted across bacteria. Note that 2 of the 62 orthologs originally included in Coleman2021 correspond to K04485, and were both discarded. For each of the remaining 32 genes, we built a gene tree using IQ-Tree using the model LG + G + I, based on which we further removed 1) 2 genes that did not show monophyly between mitochondria and α-proteobacteria, indicating unresolved paralogy and 2) 11 genes where mitochondria/α-proteobacteria were placed at a basal position to all other bacteria with different rooting approaches, indicating fast-evolving genes. This returned 19 mitochondrial (some encoded by the nuclear genome) genes conserved across the bacterial tree, which were used in the main molecular clock analysis (Supplementary Table S3 and Supplementary Fig. S1).

### Ancestral Lifestyle Reconstruction

The 16S rRNA genes of each analyzed symbiont group were collected from the NCBI Entrez Nucleotide database with the keywords “taxon_name[ORGN] AND 16S[TITL] AND rrna AND 1000:2000[SLEN] NOT spacer NOT trna” (April 2022). We also retrieved 16S rRNA genes predicted by RNAmmer v1.2 (Lagesen et al. 2007) from genomes deposited in Genbank (August 2021). Those without any information of host or isolation source in the metadata were removed (see Supplementary Materials). All remaining sequences were clustered using CD-HIT v4.7 (Li and Godzik 2006) with 98.7% as the identity cutoff (Yarza et al. 2014) into operational taxonomic units (OTUs), and 1 representative was chosen from each OTU (see Supplementary Materials for alternative strategies in sequence selection). Phylogenies were then reconstructed using IQ-Tree v2.1.3 with the model GTR + G + I based on 16S rRNA alignment performed by MAFFT v7.22261. We applied the stochastic character mapping (SCM) (Huelsenbeck et al. 2003) to directly sample from the joint posterior distribution, using *make.simmap* from the R package “phytools” v0.2-40 (Revell 2024) to estimate ancestral hosts (or lifestyles) of modern symbionts. To account for phylogenetic uncertainty, we applied the so-called most-recent-common-ancestor approach (Pagel et al. 2004) by identifying the last common ancestor (LCA) of the group of species of interest, reconstructing the state of that node, and then summarizing the information across 100 ultrafast bootstrap (UFB) trees reconstructed by IQ-Tree (settings: -m GTR + G). For each of the 100 UFB trees, 100 simulated stochastic character maps were generated using the MCMC (Markov chain Monte Carlo) algorithm implemented in phytools under the “all-rates different” model which allows transition rates at all directions to be different, totaling 100×100=10,000 simulations. All ancestral state reconstruction (ASR) analyses were performed on phylogram—a phylogeny where branch lengths indicate the expected number of substitutions per site, built by the 16S rRNA gene, as done in previous studies (Wang and Luo 2021).

Specifically, let $S_{i}$ be the inferred lifestyle (or state) of the *i*th internal node of symbionts, which can take any possible hosts observed in the group of modern symbionts (including free living), denoted as $s_{i}$. By SCM, the marginal probability that the *i*th internal node of symbionts takes the state $s_{i}$ may be approximated by


$$\begin{array}{l} \begin{array}{l} P\left(S_{i}=s_{i}\right)\approx \frac{n_{\left\{S_{i}=s_{i}\right\}}}{n},\; \end{array} \end{array}$$ (1)

where $n_{\left\{S_{i}=s_{i}\right\}}$ is the number of times that node *i* takes the state $s_{i}$ during *n* rounds of SCMs. Likewise, the same approach can be extended to estimate the joint probability that internal nodes 1 to *k* take states $s_{1},\ldots ,s_{k}$, respectively by


$$\begin{array}{l} \begin{array}{l} P\left(S_{1}=s_{1},\ldots ,S_{k}=s_{k}\right)\approx \frac{n_{\left\{S_{1}=s_{1},\ldots ,S_{k}=s_{k}\right\}}}{n},\; \end{array} \end{array}$$ (2)

where $n_{\left\{S_{1}=s_{1},\ldots ,S_{k}=s_{k}\right\}}$ is the number that the event $\left\{S_{1}=s_{1},\ldots ,S_{k}=s_{k}\right\}$ happens during all of the *n* rounds of SCMs. More details of the SCM-based ASR inference are given in Supplementary Note S3.1.2.

### pRTC (probability-based RTC) Informed by Host-Symbiont Association

Denote the data (i.e., sequence alignments, which in the present study denote the concatenated gene alignment) for molecular dating as *D*, and the divergence times as $t=\left(t_{1},\ldots ,t_{k}\right)$ where $t_{1},\ldots ,t_{k}$ indicate the divergence time of the *k* ancestral nodes of modern symbionts. Let $S_{i}$ be the inferred lifestyle (or state) of the *i*th ancestral node of symbionts, which can take any possible hosts observed in the group of modern symbionts or free-living, denoted as $s_{i}$. Let *A* indicate the event that the origin time of all symbionts is no earlier than that of their host, that is, $A=\left\{t_{1}\leq t_{S_{1}},\ldots ,t_{k}\leq t_{S_{k}}\right\}$. It follows that


$$\begin{array}{l} \begin{array}{l} \begin{array}{lll} & f\left(t|D,A\right)\propto f\left(t|D\right)P\left(A|t,D\right)\; & \;\;\;\;\;=f(t|D)P(A|t)\; \end{array}\; \end{array}\begin{array}{l} \; \end{array} \end{array}$$ (3)

where f(t|D,A) is the posterior of divergence times *t* given sequence data *D* and the assumption *A*, f(t|D) is the posterior of divergence times *t* given only sequence data *D*, and P(A|t) indicates the probability *A* is satisfied given time *t*. We further assume that the inference of ancestral lifestyles of modern symbionts depends on only the phylogenetic comparative data estimated by SCM and is independent of time, that is, $P\left(S_{1}=s_{1},\ldots ,S_{k}=s_{k}|t\right)=P\left(S_{1}=s_{1},\ldots ,S_{k}=s_{k}\right)$. The second term on the right-hand side of Eq. (3) can be rewritten as


$$\begin{array}{llllll} & P\left(A|t\right)=P\left(t_{1}\leq t_{S_{1}},\ldots ,t_{k}\leq t_{S_{k}}|t\right)\; & =\underset{s_{1},..,s_{k}}{\sum }\;P\left(t_{1}\leq t_{S_{1}},\ldots ,t_{k}\leq t_{S_{k}}|t,S_{1}=s_{1},\ldots ,S_{k}=s_{k}\right)\; & \;P\left(S_{1}=s_{1},\ldots ,S_{k}=s_{k}|t\right)\; & =\underset{s_{1},..,s_{k}}{\sum }\;P\left(t_{1}\leq t_{s_{1}},\ldots ,t_{k}\leq t_{s_{k}}|t,s_{1},\ldots ,s_{k}\right)P\left(S_{1}=s_{1},\ldots ,S_{k}=s_{k}\right)\; & =\underset{s_{1},..,s_{k}}{\sum }\;\left(\underset{i=1}{\overset{k}{\prod }}\;I\left(t_{i},t_{s_{i}}\right)\right)P\left(S_{1}=s_{1},\ldots ,S_{k}=s_{k}\right),\; \end{array}$$

where $I\left(t_{i},t_{s_{i}}\right)$ is defined as


$$\begin{array}{l} I\left(t_{i},t_{s_{i}}\right)=\left\{ \begin{array}{l} 1,\;t_{i}\leq t_{s_{i}}\;0,\;t_{i}>t_{s_{i}}\; \end{array}\right.. \end{array}$$

Particularly, we define $I\left(t_{i},t_{fl}\right)=1\;$ (free-living ancestral state) since the RTC that symbiont’s age is no older than the host’s is automatically satisfied for free-living bacteria.

Furthermore, assuming that the states of the ancestral nodes of symbiotic bacteria are independent of each other so that $P\left(S_{1}=s_{1},\ldots ,S_{k}=s_{k}\right)=\prod {}_{i=1}^{k}\;P\left(S_{i}=s_{i}\right)$, which generally holds if the ancestral nodes are not phylogenetically too closely related (see Supplementary Fig. S2b for details), the above can be rewritten as


$$\begin{array}{l} \begin{array}{l} P\left(A|t\right)=\underset{i=1}{\overset{k}{\prod }}\;\underset{s_{i}}{\sum }\;I\left(t_{i},t_{s_{i}}\right)P\left(S_{i}=s_{i}\right).\; \end{array} \end{array}$$ (4)

Collectively, by introducing Eq. (4) into Eq. (3), we have


$$\begin{array}{l} \begin{array}{l} f\left(t|D,A\right)\propto f\left(t|D\right)\left[\underset{i=1}{\overset{k}{\prod }}\;\underset{s_{i}}{\sum }\;I\left(t_{i},t_{s_{i}}\right)P\left(S_{i}=s_{i}\right)\right].\; \end{array} \end{array}$$ (5)

The above indicates that estimating the posterior time estimates under the assumption that the origin time of all symbionts is no earlier than their hosts’ while considering the uncertainty in the inference of ancestral hosts can be obtained by:

i) Perform ASR to obtain the probability of each ancestral state for selected nodes in the group of modern symbionts of interest.
ii) Perform Bayesian molecular clock-dating analysis with fossil calibrations but without any RTC.
iii) For each MCMC posterior sample collected from Step 2), perform a rejection sampling by accepting it with a probability according to Eq. (5) (based on the ASR results obtained from Step 1).

Obviously, Step 2 is independent of Step 1, and Step 3 is based on Step 1 and Step 2.

### Simulation-Based Comparison of Time Estimates With and Without pRTC

To show that the host-bacteria association-informed pRTC framework can improve divergence time estimation, we conducted a number of simulations. We first simulated a timetree with 26 tips, 20 bacteria, 5 eukaryotes, and 1 outgroup. The ages of the root and the origin time of the host (i.e., the LCA of euk1 and euk2 in Supplementary Figs. S3 and S4) were fixed as 2.0 Ga and 1.01 Ga, respectively. The bacterial subtree (cyan clades in Supplementary Fig. S3) was simulated under a birth-death process (Yang and Rannala 1997) (birth rate = 0.4, death rate = 0.2 [lineages/100 Ma], sampling proportion = 0.1; see also Supplementary Note S4.1) with the LCA of bacteria occurring at 1.0 Ga (Supplementary Fig. S3a, d–f), 0.8 Ga (Supplementary Fig. S3b), or 0.6 Ga (Supplementary Fig. S3c). Assuming the transition between 2 lifestyles (states), host-associated and free living, next we used phytools v0.2-40 (Revell 2024) to simulate lifestyle evolution starting from a host-associated LCA of the bacterial clade under 3 different rate matrices, $Q=\left(\begin{array}{lll} -1 & \;1\;1 & -1\; \end{array}\right)$ (Supplementary Fig. S3a–c, 3f), $Q=\left(\begin{array}{lll} -5 & \;5\;5 & -5\; \end{array}\right)$ (Supplementary Fig. S3e), and $Q=\left(\begin{array}{lll} -10 & \;10\;10 & -10\; \end{array}\right)$ (Supplementary Fig. S3f). We performed 20 ASR analyses to reconstruct the ancestral lifestyle of each internal node in the bacterial clade, and the result was averaged. The probability that the LCA of the bacterial clade takes a host-associated or a free-living lifestyle was later used in the pRTC framework whose divergence time was forced to be younger than that of the host with a certain probability (for details see the previous section). Furthermore, we employed AliSim (Ly-Trong et al. 2022) to simulate alignments of 200 amino acids under the substitution model LG + G (Supplementary Fig. S3a–e) or LG + G + C20 (Supplementary Fig. S3f) with an independent relaxed-clock model (see Supplementary Note S4.1). The above was repeated 30 times to generate 30 data sets under each of the 6 situations that is, the 6 panels in Supplementary Fig. S3. For each data set, time estimation was performed both with (boxplots in green in Supplementary Fig. S4) and without pRTC (blue) to compare their results based on the difference in the time estimates from the true values (those in the simulated timetree). Note that, for simplicity, the lifestyle and sequences were simulated independently. However, in real cases, they may not be independent, which would be worth exploring in future studies.

### Bayesian Sequential Approach to Dating the Bacterial Tree of Life

We applied a Bayesian sequential approach (dos Reis et al. 2012; Álvarez-Carretero et al. 2022) to establish a timeline of the bacterial tree, which allows using the genome-scale data and abundant fossil-based calibrations of eukaryotes to better constrain symbionts’ ages through the pRTC framework (see Supplementary Note S1 for details). The Bayesian sequential approach for clock-dating analysis consists of: 1) running a dating analysis of the first subset of data and collect the posterior samples of times and rates, 2) approximating the estimated posterior time densities in the first-step analysis by a parametric probability distribution and use it as the prior in a subsequent analysis with a second data subset with different genes from the first one (see Supplementary Materials for details). We first used the PAML dating program MCMCtree (PAML v4.10.0, Yang 2007) (see Supplementary Note S1.2 for details) to date a eukaryote tree with 29 selected species based on our previous study (Wang and Luo 2021) and 320 orthologs (Strassert et al. 2021) with 11 fossil-based calibrations (Supplementary Note S3.2). Then, we fitted ST, SN, and Gamma (G) distributions to the posterior time densities inferred with this first data subset so that we could use them as priors to calibrate the matching eukaryotic nodes in the tree topology fixed during the second part of the analysis (i.e., bacterial tree with a eukaryotic subtree). Note that the 2 data subsets were non-overlapping (i.e., different genes between the first and the second data subsets).

Together with the fitted skew-t (ST), skew-normal (SN), and G distributions, we used the following bacterial calibrations to constrain the node ages in the second part of the Bayesian sequential dating approach (Supplementary Note S3.2). The calibrations from within bacteria were the 1.6 Ga-old akinete fossils for the filamentous cyanobacteria Nostocales (Tomitani et al. 2006), the 1.7 Ga-old microfossils for the coccoid cyanobacteria order Pleurocapsales (Zhang and Golubic 1987), the GOE (Great Oxidation Event) ~2.32 Ga for photosynthetic cyanobacteria (Luo et al. 2016), and the hydrocarbon biomarkers for green and purple sulphur bacteria both dated at 1.64 Ga (Brocks et al. 2005). We used the maximum age of the aforementioned calibrations, 2.32 Ga, to constrain the minimum age of the root. If not otherwise specified, in all molecular clock analyses we followed Betts et al. 2018 to apply a uniform distribution as the prior: a soft tail was applied to the upper calibration bound, which means a small probability (by default 2.5%) where the age is beyond the bound, and a hard bound was set to further constrain minimum age, meaning that the age cannot be lower than the bound. This makes sense as minimum bounds are based on solid fossil or biomarker records while the maxima are often based on indirect evidence (Benton and Donoghue 2007; Marshall 2019).

### A Bootstrap-Based Approach to Improving MCMCtree Branch Length Estimate by Incorporating the Mixture Substitution Model

We used the approximate likelihood calculation implemented in MCMCtree (dos Reis and Yang 2011) to speed up timetree inference. Specifically, CODEML, will be called to first compute the branch lengths, the Hessian, and the gradient, which are then used by MCMCtree to approximate the likelihood calculation. Different from previous studies which often use the LG + G substitution model, we applied the bootstrap-based method developed in the present study to integrate the site-heterogeneous profile mixture model LG + G + C60 (Quang et al. 2008) implemented in IQ-Tree when calculating the branch lengths, the Hessian, and the gradient with CODEML. We based our method on the assumption that the maximum likelihood (ML) estimates of branch length follow a multivariate normal (MVN) distribution, according to the ML estimation theory stating that ML estimators are asymptotically multivariate normally distributed (Pawitan 2003). The idea was originally proposed by Thorne et al. 1998 and later implemented in PAML-MCMCtree as the approximate likelihood method (dos Reis and Yang 2011). This approach first obtains the maximum-likelihood estimates (MLEs) of the branch lengths $\hat{\theta }$ = $\left({\hat{\theta }}_{1},\;\ldots ,{\hat{\theta }}_{n}\right){}^{T}$, plus the gradient vector **g** and the Hessian matrix **H**, which are the first and second derivatives of the log-likelihood evaluated at $\hat{\theta \;}$, respectively. Next, according to Eq. (1) in dos Reis and Yang (2011), the Taylor expansion at the second-order derivative was applied to approximate the log-likelihood


$$\begin{array}{l} \ell (\theta )=\ell (\overset{}{\hat{\theta }})+g^{T}△\theta +\frac{1}{2}△\theta^{T}H△\theta \end{array}$$ (6)

where θ  = $\left(\theta_{1},\;\theta_{2},\;\ldots \;,\theta_{n}\right){}^{T}$ denotes the parameters to estimate (branch lengths) and ℓ(θ) is the log-likelihood as a function of branch lengths.

While the gradient is zero at the ML estimate (g=0), directly calculating **H** under IQ-Tree’s profile mixture and other complex substitution model is difficult and has not yet been implemented in the program. However, note that when the sample size is large enough, the Hessian matrix evaluated at $\hat{\theta }$ is approximately the negative inverse matrix of the covariance matrix of the MLEs $V\left(\hat{\theta }\right)$ (Pawitan 2003):


$$\begin{array}{l} H\approx -V{\left(\hat{\theta }\right)}^{-1}. \end{array}$$ (7)

Hence, we instead applied a bootstrap method to estimate $V\left(\hat{\theta }\right)$ by the bootstrap covariance matrix $\hat{V}\left({\hat{\theta }}^{*}\right)$ (Efron 1982) by taking *B* bootstrap alignments and estimating the MLEs of branch lengths ${\hat{\theta }}^{*}$:


$$\begin{array}{l} \begin{array}{l} V\left(\hat{\theta }\right)\approx \hat{V}\left({\hat{\theta }}^{*}\right)=\frac{1}{B-1}\overset{B}{\underset{b=1}{\sum }}({\hat{\theta }}^{\left(b\right)}-\bar{{\hat{\theta }}^{*}}){\left({\hat{\theta }}^{\left(b\right)}-\bar{{\hat{\theta }}^{*}}\right)}^{T}\; \end{array} \end{array}$$ (8)

where ${\hat{\theta }}^{\left(b\right)}$ indicate the MLEs of the branch lengths in the *b*th bootstrap sample. Specifically, 1000 bootstraps (*B* = 1000) were performed using the site-heterogeneous profile mixture model LG + G + Cxx (Cxx represents C10–C60) with a fixed tree topology by IQ-Tree, therefore generating 1000 bootstrap MLEs for each branch length and allowing calculating the bootstrap covariance matrix $\hat{V}\left({\hat{\theta }}^{*}\right)$. Accordingly, the Hessian matrix can be approximated by solving the inverse matrix of $-\hat{V}\left({\hat{\theta }}^{*}\right)$. We obtained the MLEs of branch lengths, the gradient as a zero matrix, and the Hessian matrix approximated by the above procedure, and generated the “in.BV” file required by MCMCtree to approximate the likelihood calculation (dos Reis and Yang 2011). A series of simulations were performed to show the feasibility of this approach to integrating profile mixture substitution model into timetree inference analyses with MCMCtree (Supplementary Note S4).

### Selection of the Best-Fitting Clock Model

We employed the mcmc3r package (dos Reis 2017; dos Reis et al. 2018) to determine the best-fitting clock models. This method can be used to find the best-fitting clock model by calculating the marginal likelihood using either the stepping-stone method (Xie et al. 2011) or the thermodynamic integration (Lartillot and Philippe 2006; Lepage et al. 2007). Specifically, we tested all the available clock models implemented in MCMCtree: the auto-correlated rate (AR; also known as the geometric Brownian motion), the independent-rate (IR) log-normal, and the strict (STR) clock models. Note that the exact likelihood calculation implemented in MCMCtree is required to collect the samples for each power posterior (McGowen et al. 2020) that the mcmc3r R package will later use to estimate the marginal likelihood with which Bayes factors and posterior probabilities can be computed. Note that the exact likelihood method implemented in mcmc3r works on only nucleotide sequences. We therefore recoded the amino acid sequences into 4-character states based on the Dayhoff-4 scheme (McGowen et al. 2020; Wang and Luo 2021) and proceeded to run MCMCtree and, subsequently, mcmc3r as aforementioned.

In the first-step sequential analysis, that is, dating the eukaryote tree based on the 320 orthologs identified in (Strassert et al. 2021), the IR and AR models were favored by similar proportions of genes (53% vs. 47%; *eukaryotic_tree* in Supplementary Fig. S5). Because posterior time estimates obtained by IR and AR were similar, we arbitrarily chose the IR model. For the second-step analysis, that is, dating the bacterial tree (148 species), we followed prior studies (McGowen et al. 2020; Wang and Luo 2021) to overcome the huge computational burden: 1) we compiled 2 reduced data sets by randomly selecting 20 and 40 species and 2) we fixed the root prior at 4.0 Ga and applied only a single internal calibration that constrained the age of the LCA of eukaryotes to 1.7–1.5 Ga (here, we did not want to estimate divergence times but simply select a better-fitting model). The AR model was shown to have the higher marginal likelihood for 69% and 73% of the genes for the 20- and 40-species sets (Supplementary Fig. S5), respectively, and was therefore selected as the preferred model, as done in a previous study (McGowen et al. 2020).

### Selection of Genes With High Verticality or Low Among-Branch Rate Heterogeneity

To test if genes used in analysis impacted the posterior dates in molecular dating of the bacterial tree, the 19 mitochondrial genes were evaluated with the so-called ∆ LL (difference in log-likelihood) as a metric of marker gene verticality (i.e., if genes were vertically inherited in evolution) (Moody et al. 2022), and relative rate difference which measures the extent of the violation of molecular clock for a gene (Wang and Luo 2021). Specifically, ∆ LL calculates the difference in likelihood in log scale between a constrained ML tree based on the topology of the species tree and the ML gene tree. Genes with a higher ∆ LL indicate large topology difference between the gene tree and the species tree, thus lower verticality (Moody et al. 2022). To this end, ∆ LL well serves as a proxy for the extent to which the gene tree differs from the species tree. The relative difference in rate between mitochondria and others was calculated as $\frac{\left|r_{mito}-r_{bac}\right|}{\max\left(r_{mito},\;\;\;\;r_{bac}\right)}$ (Wang and Luo 2021), where the substitution rates of mitochondria or bacteria lineages were estimated by CODEML from PAML (Yang 2007). The higher the relative rate difference is, the more rate heterogeneity between lineages would be expected. Selecting only those with the smallest relative rate difference in molecular dating could ensure less violation of the molecular clock caused by the fast-evolving mitochondrial lineages, as used in Wang and Luo 2021. We selected the 5, 10, and 15 top-ranking genes using each of the above 2 measures and repeated the molecular clock analyses. Additionally, we performed the analysis with the full set of 32 mitochondria-related genes.

## Results

### A New Framework for Dating Deep-Time Bacterial Evolution Informed by Ancient Bacterial Symbiosis

We propose a new framework to address the paucity of bacterial fossils in dating the bacterial tree by leveraging the temporal information of eukaryotes recorded in their abundant fossils. Briefly, by leveraging mitochondrial endosymbiosis which states that mitochondria of eukaryotes are phylogenetically affiliated with α-Proteobacteria (Martijn et al. 2018; Muñoz-Gómez et al. 2022), we incorporated eukaryotes into a bacterial phylogeny. Through this, it is tempting to co-estimate the ages of both the eukaryotic host and bacterial symbiont (Wang and Luo 2021) while applying the recently developed RTC-based molecular dating (Davín et al. 2018; Magnabosco et al. 2018; Szöllõsi et al. 2022). In the context of host-symbiont association, this means constraining the origin time of the symbiont’s crown group to be no earlier than that of the host’s total group, thereby providing maximum time constraints to symbionts whose temporal information may further be propagated across bacteria.

**Figure 1.**
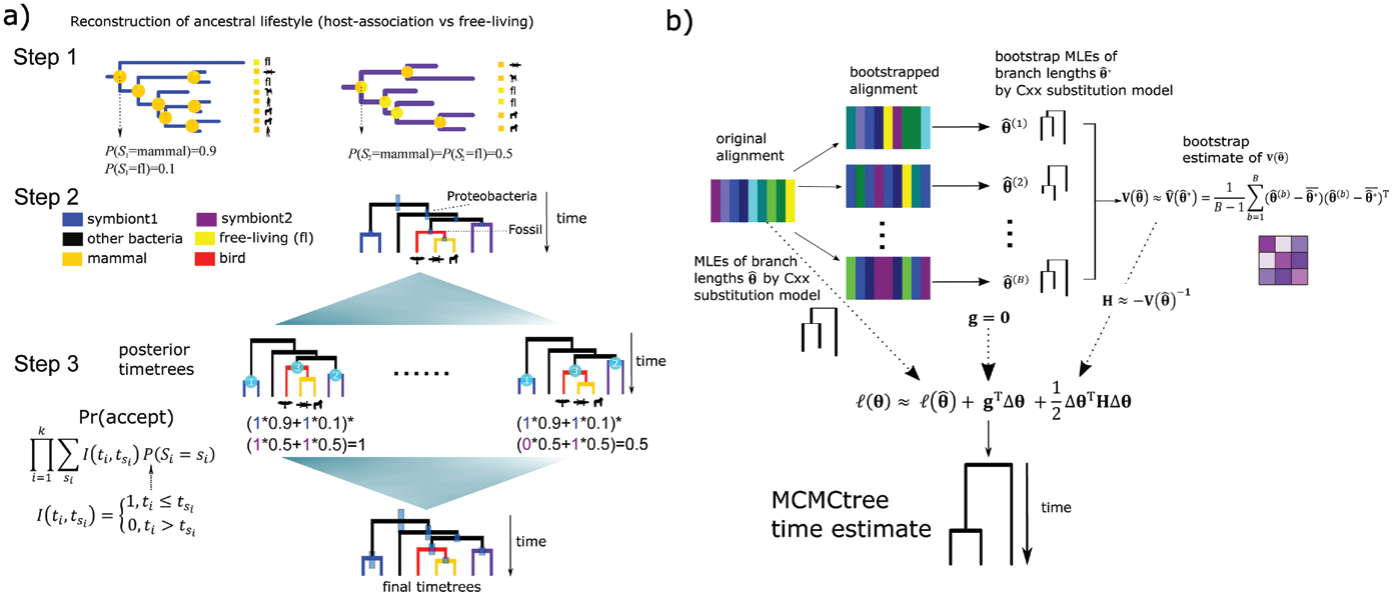
Graphical illustration of the 2 new methods in molecular dating developed in the present study. (A) Procedure of the pRTC dating informed by host-symbiont association. Suppose that there are 2 symbiotic lineages, Symbiont1 from non-Proteobacteria (blue) and Symbiont2 from α-proteobacteria (purple), and most of their members are mammal symbionts (orange) but some are free-living (yellow). First, ASR is performed on the phylogram of 16S rRNA tree to obtain the probability of each ancestral state taken by the LCA of the 2 “symbiont” clades respectively, that is, $P\left(S_{1}\right)$ and $P\left(S_{2}\right)$. Second, a phylogeny consisting of symbionts and mammals is constructed by mitochondrial genes, and MCMCtree is run with fossil calibrations but without RTCs. Third, rejection sampling is performed to remove posterior timetrees obtained in the previous step based on the requirement that symbiont’s origin must postdate the host. Specifically, we require that the age of the crown group of the symbionts (node1 and node2) to be no older than that of the total-group mammals (node3), weighted by $P\left(S_{i}=mammal\right)$. The use of mammals’ total group is because there could be stem-group mammals that are unsampled or go extinct but that have Symbiont1 or Symbiont2 as symbiont. For the free-living state, we define $I\left(t_{i},t_{fl}\right)=1\;$ as the RTC is automatically satisfied for free-living bacteria (see Methods). In the final timetree, the divergence time of symbiont lineages becomes younger than that inferred before host association is considered, suggesting that incorporating RTC informed by host association improves the molecular clock inferences. The icons of animals are obtained from PhyloPic (https://www.phylopic.org/) under CC0 1.0 Universal. (B) Overflow of the bootstrap-based approach to incorperating branch length estimates inferred under site-heterogeneous profile mixture substitution models such as LG + G + Cxx where xx indicates 10–60 categories of equilibrium frequency. θ = ${\left(\theta_{1},\;\theta_{2},\;\ldots ,\theta_{n}\right)}^{T}1187{\left(\theta_{1},\;\theta_{2},\;\ldots ,\theta_{n}\right)}^{T}$ denotes the parameters to estimate (branch length, i.e., expected number of amino acid substitutions per site) and l(θ) is the log-likelihood as a function of branch lengths. Briefly, the MLE of branch length $\hat{\theta \;}$ is obtained by LG + G + Cxx, and the bootstrap covariance matrix of branch lengths $V\left({\hat{\theta \;}}^{*}\right)\;$ is calculated to approximate the Hessian matrix (second-order derivative of the log-likelihood of branch length), which are further used for time estimation by MCMCtree.

It is well possible that the ancestor of modern symbionts adapted to a different host or even a free-living lifestyle. We formulate a 3-step approach accounting for the possibility of host shift of symbionts through ASR and fit it into RTC-based dating in a probabilistic framework, which we call probability-based RTC (pRTC; see Methods for details). First, ASR is performed to estimate the probabilities that the ancestral nodes of modern symbionts are associated with different hosts or are free-living. Second, Bayesian clock dating is performed using mitochondria-associated genes with fossil calibrations but without RTC to infer posterior time estimates where the ages of symbionts and eukaryotic hosts (placed as the mitochondrial clade sister to α-proteobacteria; see also above) are co-estimated. Third, the posterior timetrees collected in Step 2 are examined to remove those where the host’s age is younger than the symbiont’s, weighted by the probability of different states (hosts) taken by the ancestor of modern symbionts estimated in Step 1, using a rejection sampling approach. The idea is further illustrated in Fig. 1a. Assume that ASR infers the ancestral lifestyles of “symbiont1” as $P\left(S_{1}=mammal\right)=0.9$ and $\;P\left(S_{1}=freeliving\right)=0.1$, and for “symbiont2” as $P\left(S_{2}=mammal\right)=0.5$ and $\;P\left(S_{2}=freeliving\right)=0.5$. For the 2 timetrees in the posterior samples shown in Fig. 1a, the probabilities of acceptance are  (0.9×1+0.1×1)×(0.5×1+0.5×1)=1, and (0.9×1+0.1×1)×(0.5×0+0.5×1)=0.5, respectively (see Eqs. 3–5 in Methods). If the above procedure is performed for all posterior timetrees collected from Step 2, the posterior time estimates of all bacterial lineages, particularly the symbiont, might shift toward the present due to the use of RTC informed by host-symbiont association (Fig. 1a). As ancient bacterial symbionts are distributed in different deep-branching lineages (Toft and Andersson 2010; McCutcheon and Moran 2012), this method has a great potential to help date the bacterial tree of life by including additional relatively ancient time constraints.

We further illustrate this idea by carrying out various simulations (see Methods; Supplementary Figs. S3 and S4). We simulated a species tree resembling the mito-endosymbiosis-based strategy mentioned above where the mitochondrial lineages (eukaryotes) are sister to α-proteobacteria (see Methods), and fixed the ages of the root and the LCA of the host at 2.0 Ga and 1.0 Ga, respectively. We simulated the sequence alignments, conducted ASR inference based on simulated lifestyle evolution, and used MCMCtree for timetree inference before and after pRTC. In all analyses, we showed that including pRTC (light green boxplots in Supplementary Fig. S4) significantly improved time estimates (paired *t*-tests; *P*-values < 0.001 in all comparisons; Supplementary Fig. S4). When the host clade was calibrated (boxplots on the right-hand side in Supplementary Fig. S4), the pattern became stronger, suggesting that estimating a more accurate divergence time for the host can improve divergence time estimation for the rest of the bacterial nodes (i.e., higher accuracy) within the pRTC framework. When the “true” age of the symbiont was gradually reduced from 1.0 Ga to 0.6 Ga, making it progressively younger than the host, the improvement in time estimates provided by pRTC decreased (panels A, B, and C in Supplementary Figs. S3 and S4). Additionally, when the lifestyle was simulated to change more rapidly during evolution so that the phylogenetic signal was weakened, the improvement in time estimates became less pronounced, probably because the ASR inference is less accurate in such cases (panels A, D, and E in Supplementary Figs. S3 and S4).

### Integrating Eukaryote Temporal Information by a Bayesian Sequential Approach

We reconstructed the phylogeny of bacteria using the 265 genomes and 60 gene markers carefully selected in a comprehensive phylogenomics study (Coleman et al. 2021) using the best-fitting site-heterogeneous profile mixture model LG + G + C60 (Supplementary Table S1a and Supplementary Note S1). We further applied the time-nonreversible model (Naser-Khdour et al. 2022) which infers the root of the bacterial tree as part of ML phylogenetic reconstruction where different root positions may have different likelihoods. This method does not depend on any outgroup, thereby avoiding long-branch attraction artifacts caused by using highly divergent sequences to root deep phylogenies. Our analyses rooted the bacterial phylogeny in the neighborhood of a clade consisting of Fusobacteriota and DST (Deinococcota, Synergistota, and Thermotogota). The analyses also supported the division of most bacterial lineages into Gracilicutes and Terrabacteria (Taib et al. 2020), and placed the candidate phyla radiation (CPR) (Patescibacteria) clade (Supplementary Fig. S6), initially suggested as the earliest-split bacterial group (Hug et al. 2016; Zhu et al. 2019), as a late-split clade sister to Chloroflexi (Supplementary Fig. S6, Supplementary Table S2, and Supplementary Note S2). This is generally consistent with a recent study that roots the bacterial tree using an independent method based on gene-species tree reconciliation (Coleman et al. 2021).

To apply pRTC based on host-symbiont co-evolution, we integrated eukaryotes and bacteria in a unified framework to co-estimate their times using the mito-endosymbiosis-based dating strategy (Wang and Luo 2021). As noted, the ages of symbionts are more precisely and accurately estimated if the host date is better constrained, thus with less uncertainty. We therefore used the genome-scale data of eukaryotes by employing a Bayesian sequential approach in which the posterior times estimated with a data set with few taxa and many genes are estimated to serve as the time priors of a second analysis with many organisms but fewer genes (dos Reis et al. 2012; Álvarez-Carretero et al. 2022). Note that the Bayesian sequential clock-dating approach is conceptually distinct from secondary calibrations. In Bayesian sequential clock dating, the posterior time distributions obtained with the first data set are subsequently used as prior distributions with the second data set (fewer and different genes from the first data set but more taxa, including those taxa used in the first data set), which helps accommodate the uncertainty in divergence times estimation from 1 analysis to another. Here the 2 data sets must be independent (i.e., different genes in data set 1 and data set 2). In contrast, dating with secondary calibrations typically relies on point estimates of divergence times (though approximated time distributions can also be used) derived from other studies, and it often employs genes overlapping with those used to obtain the original calibrations from those studies (dos Reis et al. 2018). If the same genes are present in both data sets, the likelihood will be squared, and therefore its calculation during the MCMC will be wrong; hence the importance of independent data sets (Álvarez-Carretero et al. 2022) (Supplementary Note S1.2). We estimated the divergence times of 29 representative eukaryotes with 11 fossil calibrations (Supplementary Figs. S7 and S8) and 320 orthologs conserved across eukaryotes (Strassert et al. 2021). The posterior time estimates of eukaryote nodes were generally in agreement with most studies (Parfrey et al. 2011; Betts et al. 2018; Gibson et al. 2018; Wang and Luo 2021) (see Supplementary Note S5 for additional discussion). Next, we fitted ST, SN, or G distributions (Supplementary Fig. S9 and Supplementary Note S1.2.2) to the posterior time distributions inferred for each node in the fixed eukaryotic tree topology (first step of the Bayesian sequential clock-dating approach; “backbone” tree with 29 eukaryotic representative taxa and 320 orthologs) so that they could be used as priors to subsequently calibrate the eukaryotic nodes in the second data set with more taxa and less genes. In this second data set, the eukaryotic subtree was then placed as a sister clade to α-proteobacteria in the larger bacterial tree that was used for timetree inference during the second step of this Bayesian sequential clock-dating approach.

### A Time-Calibrated Bacterial Phylogeny Based on Ancient Symbiosis

**Figure 2.**
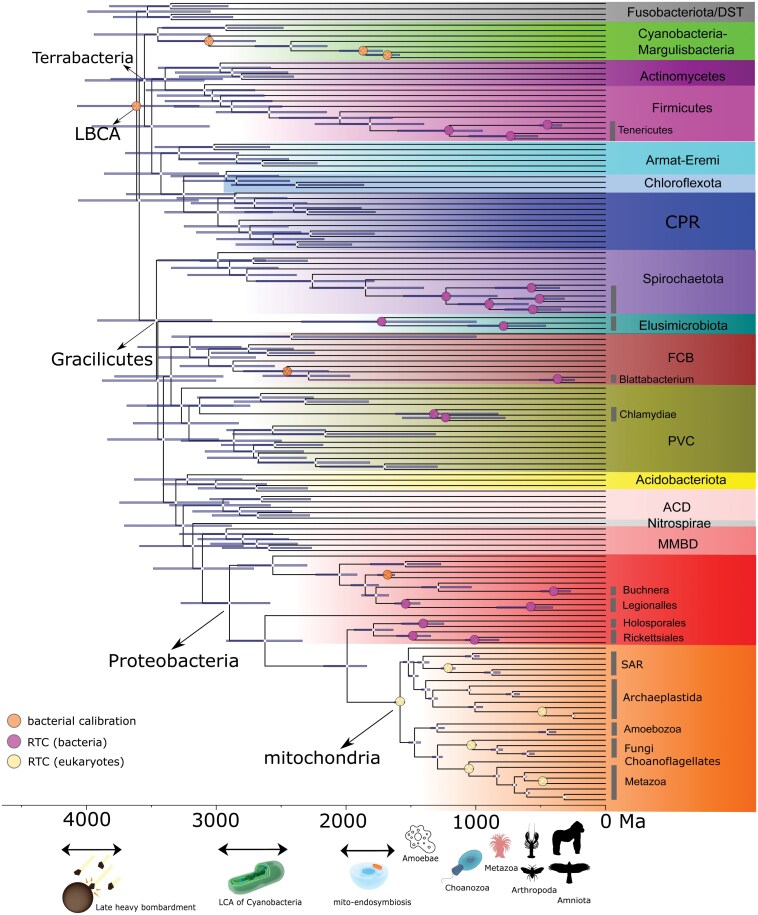
An evolutionary timeline of the bacterial tree of life established by the 3-step pRTC procedure. The root position is determined using IQ-Tree’s nonreversible substitution model in ML phylogenomic tree reconstruction using the 19 mitochondrial genes. Node bars indicate the 95% HPD interval of age estimates. Nodes with circles indicate the bacterial calibration points (including the root). Eukaryotic calibrations obtained from sequential Bayesian dating are given in Supplementary Fig. S4. RTCs of the 28 bacterial symbionts and 5 eukaryotic hosts (LCA of eukaryotes, total-group animals, total-group ciliates, total-group insects, and crown-group fungi) are denoted by circles. Because the 28 symbionts are selected from “symbiont” clades, a total of 28 − 9 = 19 internal nodes serve as the RTCs for bacteria. The organism images are placed at the origin times of eukaryotes of interest co-estimated with bacterial lineages. Detailed posterior ages for clades of interest are summarized in Supplementary Table S4. DST: Deinococcota, Synergistota, and Thermotogota; Armat-Eremi: Armatimonadetes and Eremiobacteraeota. CPR: Candidate phyla radiation; FCB: Fibrobacterota, Chlorobiota, Bacteroidota; PVC: Planctomycetota, Verrucomicrobiota, Chlamydiota, and related lineages; ACD: Aquificota, Campylobacterota, and Deferribacterota. MBDD (δ-Proteobacteria): Myxococcota, Bdellovibrionota, Desulfomonadota, and Desulfobacterota. The images of the choanozoan, and the cyanobacteria and mitochondria are credited to Urutseg and Kevin Song, respectively under CC BY-SA 3.0 (https://creativecommons.org/licenses/by-sa/3.0/ ) with slight modifications. All other organism images are distributed under CC 1.0.

To apply the pRTC dating approach based on ancient symbiosis, we first performed comprehensive MCMC-based ASR to infer the ancestral lifestyles of 7 well-known ancient clades where symbionts are widely distributed, 4 at a phylum level (Spirochaetota, Tenericutes, Chlamydiae, and Elusimicrobiota) and 3 at an order level (Rickettsiales, Holosporales, and Legionellales). The ASR was performed using the 16S rRNA gene, which best captures bacterial lifestyle diversity by including environmental and uncultured samples, based on the lifestyle information of modern members within each of the ancient bacterial clades mainly consisting of symbionts (Supplementary Figs. S10 and S11). We additionally included 2 genus-level symbionts (*Buchnera* and *Blattabacterium*) which are classical host-symbiont co-evolution examples (Perreau and Moran 2022). A total of 19 ancestral nodes consisting of 28 symbiont lineages from the above 9 symbiont clades were selected to represent the host-symbiont association-based RTCs in subsequent analyses (Fig. 2, Supplementary Fig. S10, and Supplementary Note S3.1).

Furthermore, we identified 19 genes well conserved across bacteria (Supplementary Figs. S7 and S8) from 108 genes evolutionarily associated with mitochondria (Muñoz-Gómez et al. 2022) (see also Supplementary Data S1). We performed molecular clock-dating analysis using a combination of these 19 genes and a 148-genome focal data set consisting of 29 eukaryotes, 28 symbionts, and 91 non-symbionts. The phylogeny was based on the tree reconstructed above using the nonreversible model and with eukaryotes placed as the sister to α-proteobacteria. The fitted ST, SN, and G distributions onto the posterior time densities inferred during the first step of the Bayesian sequential clock-dating approach, together with the 5 lineage-specific fossil- or biomarker-based calibrations within bacteria, were used to constrain various node ages in the bacterial tree, embedded with the eukaryotic subtree, during the second step of this timetree inference analysis. We used the approximate likelihood calculation implemented in MCMCtree (dos Reis and Yang 2011) to speed up the timetree inference given our large phylogenomic data set. However, the amino acid substitution models (e.g., LG + G) used to calculate branch lengths in PAML-MCMCtree (and many other molecular dating software such as BEAST and MrBayes) do not consider across-site compositional heterogeneity and often show poor model fit in reconstructing deep phylogenies (Gouy et al. 2015; Moody et al. 2022; Baños et al. 2024).

We therefore developed a bootstrap-based approach to incorporating branch length estimates inferred under the site-heterogeneous profile mixture substitution model LG + G + C60, which better accommodates issues in deep-time phylogenetics like substitution saturation and can be easily used by MCMCtree for timetree inference. Briefly, ML trees were built for 1000 bootstrap alignments, and the negative inverse of the bootstrap covariance matrix of their branch lengths, which can be calculated with either IQ-TREE or any other software implementing mixture models, was calculated to approximate the Hessian matrix (which describes the curvature of the log-likelihood function around the MLEs of branch lengths); note that by default the branch lengths and Hessian matrix are calculated with CODEML (or BASEML for nucleotides) under only simpler substitution models like LG + G. Simulations showed considerable improvements in the estimates of both ages and absolute rates by integrating site-heterogeneous models with this approach, particularly in the absence of internal calibrations or if the divergence times were older than 3.0 Ga (Supplementary Figs. S12 and S13; see Supplementary Note S4 for details).

Next, we followed Eq. (5) (see Methods) to apply a rejection sampling approach to filter out the inferred timetrees without applying RTC from Step 2, based on the probabilities of the ancestor of modern symbionts being associated with different eukaryotic hosts or free-living inferred from the first step in the sequential dating (Fig. 1a and Supplementary Fig. S14c). Collectively, using a carefully selected focal dating scheme, we estimated that the LBCA occurred around 3648 Ma (95% highest posterior density [HPD]) 3162–4111 Ma) (Fig. 2), followed by the split between Terrabacteria and Gracilicutes estimated to be 3629 Ma (95% HPD 3167–4103 Ma). Most major clades, many of which are beyond a phylum level, diverged during 3000–3500 Ma (Fig. 2).

**Figure 3.**
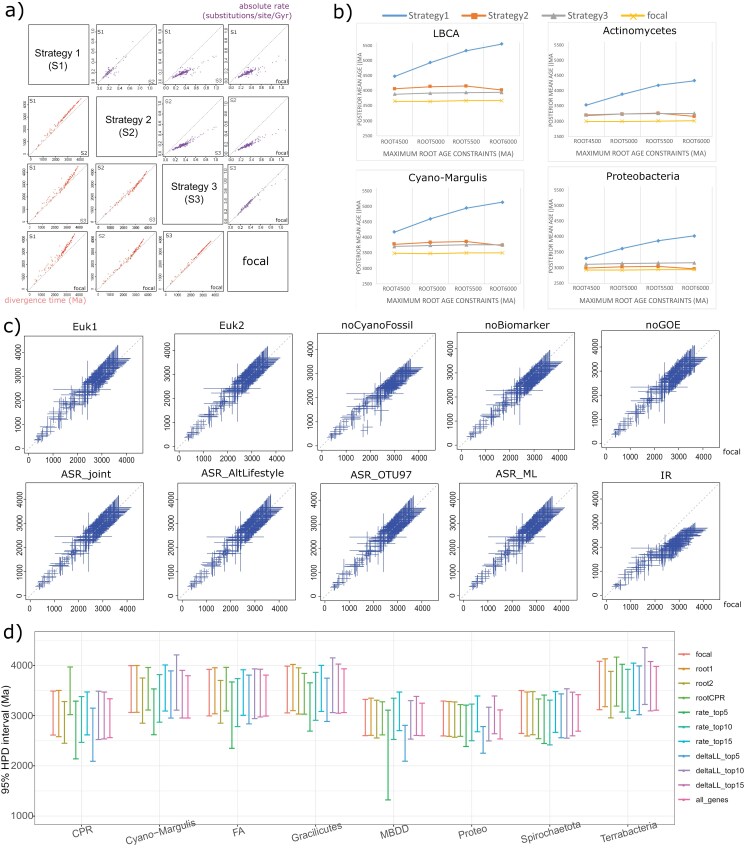
Assessing the uncertainty in Bayesian clock-dating analysis. (A) Comparison of the posterior mean ages (lower triangle; unit: Ma) and rates (upper triangle; unit: number of substitutions per site per Ga) of bacteria estimated with different strategies. *Strategy1* (traditional strategy used in prior studies): 5 bacterial calibrations with hard minimum bounds and a soft maximum bound < 4.5 Ga at LBCA, no eukaryote timing information, substitution model LG + G, no RTCs. *Strategy2*: bacterial calibrations, eukaryote times by Bayesian sequential clock-dating approach, substitution model LG + G, no RTCs. *Strategy3*: bacterial calibrations, eukaryote times by Bayesian sequential clock-dating approach, substitution model LG + G + C60, no RTCs. Focal: bacterial calibrations, eukaryote times by Bayesian sequential clock-dating approach, substitution model LG + G + C60, with RTCs. (B) Posterior mean ages of the selected clades calculated under root maximum prior ages from 4500 to 6000 Ma with the focal strategy and Strategies 1–3. (C) Divergence times of bacteria estimated by alternative schemes (alternative calibration hypotheses constraining differently the age of contentious nodes) (*y*-axis; Supplementary Data S3) versus the one in the focal strategy (*x*-axis). The bars denote the 95% HPD. *Euk1*: the minimum time bound of crown-group red algae alternatively set as 1.6 Ga based on *Rafatazmia*; *Euk2*: the minimum time bound of animal crown group alternatively set as 0.89 Ga (Supplementary Note S3.2); *noCyanoFossil*: the 2 cyanobacteria minimum time bounds (total-group Nostocales and Pleurocapsales) removed; *noBiomarker*: the 2 bacterial biomarker minimum time bounds (total-group Chromatiaceae and Chlorobi) removed; *noGOE*: the minimum time bound based on GOE removed. *Joint_prob*: rejection sampling based on calculating the joint probability (see Supplementary Fig. S6b) in Eq. (2). *Alt_lifestyle*: ASR inferred with alternative classification of lifestyles of modern symbionts (Supplementary Note S3.1). *OTU97*: ASR performed with OTU at a cutoff of 97% sequence identity. *ASR_ML*: maximum likelihood algorithm instead of MCMC used in ASR. *IR*: the independent rate model is used instead of the AR model. (D) Changes in the posterior time estimates shown as 95% HPD interval resulting from different root positions of the phylogenomics tree (*root1*, *root2*, and *rootCPR*), and the use of different genes. *rate_top* and *deltaLL_top*: the 5, 10, and 15 (best) top-ranking genes according to relative rate difference between mitochondrial and bacterial lineages or △LL which measures the degree of species- and gene-tree incongruence with *root0* as the root position (see Methods and Supplementary Data S3). *all_genes*: molecular dating performed on all 32 mitochondrial genes conserved across bacteria (also *root0* as the root position). The center is the posterior mean age. FA: Firmicutes and Actinomycetes. MBDD (δ-Proteobacteria): Myxococcota, Bdellovibrionota, Desulfomonadota, and Desulfobacterota.

We gradually included eukaryotic temporal constraints, the LG + G + C60 substitution model, and host-symbiont association-based RTCs to check the improvement of posterior time estimates of the bacterial tree of life (lower triangle in Fig. 3a). The inclusion of eukaryote’s timing (*Strategy2*: bacterial calibrations plus eukaryote time estimates by sequential molecular dating, substitution model LG + G, no RTCs) led to a 9.6 ± 4.5% (mean ± standard deviation) decrease in the posterior time estimates compared with *Strategy1* where the 5 bacterial calibrations were the only constraints (*Strategy1:* 5 bacterial calibrations with no eukaryote timing information, substitution model LG + G, no RTCs). When the branch lengths were estimated under the LG + G + C60 model (*Strategy3;* bacterial calibrations, posterior eukaryote time estimates with the sequential clock-dating approach, substitution model LG + G + C60, no RTCs), those nodes between 3000 and 2000 Ma were estimated to be older by 5.9 ± 4.4% (*Strategy2* vs. *Strategy3*). Furthermore, when pRTC was enabled under Strategy3, the estimated posterior times for almost all nodes were systematically younger (8.0 ± 5.5%, *Strategy3* vs. *Focal* in Fig. 3a; Focal: bacterial calibrations, eukaryote time estimates by sequential molecular dating, substitution model LG + G + C60, with RTCs), suggesting that the constraint on the time order imposed by host-symbiont co-evolution was propagated through the bacterial tree of life. Collectively, the combination of the use of time constraints provided by eukaryote temporal information, site-heterogeneous profile mixture models, and host-symbiont association-based RTCs estimated the divergence times of analyzed bacteria to be 14.0 ± 7.5% younger than those obtained without any of these settings (*Strategy1* vs. *Focal* in Fig. 3a [upper triangle]; please see Supplementary Note S1.2.1 for details on how to estimate the absolute rates; this represents a net reduction and some effects canceled out as illustrated above). As to the posterior rate for each branch, the use of C60 led to an almost doubling of rate estimate (upper triangle in Fig. 3a; *Strategy2* vs. *Strategy3*), indicating that the absolute rates are largely underestimated by the compositionally homogeneous LG + G model. Moreover, when we successively increased the maximum time prior of the root from 4500 Ma to 6000 Ma, the inferred posterior times of bacterial lineages were ~20% older under *Strategy1*, but remained almost unchanged with the focal, *Strategy2,* and *Strategy3* schemes (Fig. 3b and Supplementary Fig. S15a), indicating that the time constraints imposed by eukaryotes effectively constrained the timing of bacterial evolution, such that their time estimates were robust to the root calibration where much debate has arisen (Nisbet and Sleep 2001; Moody et al. 2022). The time estimates obtained by the focal scheme were consistently younger than those estimated by *Strategy3* (Fig. 3b), suggesting that the pRTC effectively constrained the divergence times.

### Accommodating the Uncertainty in Divergence Time Estimation

We evaluated the impact that multiple sources of uncertainty can have on species’ divergence times estimation. Firstly, fossil-based calibrations are known to play a major role in molecular dating (Ho and Duchêne 2014). We used a total of 17 calibrations: 11 to constrain node ages in the eukaryotic clades, 5 within bacterial clades, and a last calibration to constrain the root age. Replacing the 1.047 Ga-old fossil of *Bangiomorpha*, the most widely recognized oldest crown-group eukaryote (Betts et al. 2018; Gibson et al. 2018), with the recently discovered but disputed 1.6 Ga-old *Rafatazmia* (Bengtson et al. 2017) (see also Supplementary Note S3.2), pushed the LCA of eukaryotes from ~1600 Ma to ~2100 Ma (Supplementary Fig. S8), but had little impact on the time estimates of bacteria (*Euk1* in Fig. 3c and Supplementary Data S2). A similar pattern was observed when the recently discovered but disputed 0.89 Ga-old sponge fossil instead of the 0.55 Ga-old one (Supplementary Note S3.2) was alternatively used as the minimum age of the total-group animals (*Euk2* in Fig. 3c). Applying other eukaryotes’ calibrations also showed very little changes to the posterior date estimates in the bacterial timetree (*Euk3-Euk5* in Supplementary Fig. S15 and Supplementary Data S3). Regarding the 5 bacterial calibrations, the 2 cyanobacteria fossils and the 2 biomarkers displayed considerable impacts on the posterior divergence time estimates as their removal led to a younger time estimate (7% on average) (*noCyanoFossil* and *noBiomarker* [*y*-axis] compared with the focal strategy [*x*-axis] in Fig. 3c and Supplementary Data S3). Replacing the 1.6 Ga-old Nostocales fossil calibration (Tomitani et al. 2006) by a more disputable estimate based on the 2.0 Ga-old fossil (Amard and Bertrand-Sarfati 1997) led to older posterior time estimates by more than ~10% (Supplementary Fig. S12b and Supplementary Data S3). Except the above, the use of all alternative fossil calibrations returned similar time estimates of both eukaryotes and bacteria (Supplementary Figs. S15b and S16).

Secondly, another source of time constraint is the use of ASR-based RTC. In the above analysis, the ancestral states (hosts) between nodes within the same tree were considered independent of each other such that their joint probability can be approximated by the multiplied marginal probability (Supplementary Fig. S2b). To check if this approximation affected the final time estimates, we directly estimated the joint probability of their ancestral states using an SCM method (see Methods). This generally did not change the posterior time estimates (*joint_prob* in Fig. 3c). Further, while we have conducted comprehensive work in classifying the traits into different hosts or free-living, there exist different interpretations of the phenotypic traits, particularly free-living, which might affect ASR. Analysis with alternative host/lifestyle classification (Supplementary Note S3.1.3) suggested little changes in posterior time estimates (*ASR_AltLifestyle* in Fig. 3c). Additionally, we tried different cutoffs of 16S OTU and a different algorithm (ML) in ASR, all of which resulted in highly consistent time estimates (Fig. 3c). Including 13 more symbiont lineages returned similar time estimates (*more_RTCs* in Supplementary Fig. S15), suggesting that adding host association-based RTCs would not lead to further change in the posterior ages of bacteria.

Thirdly, dependence of the results on the phylogenomic tree topology was investigated. When timetree inference analysis was performed with 3 alternative root positions, 2 near the root (*root0*) used in the focal analysis (*root1* and *root2* in Fig. 3d and Supplementary Fig. S6), and 1 (*rootCPR*) at the CPR clade as suggested by earlier studies (Hug et al. 2016; Zhu et al. 2019), the posterior time estimates remained similar for most clades, except those that were alternatively placed as an early-split phylogenetic position, particularly the CPR. Alternative phylogenetic positions of mitochondria or between eukaryotic groups (Supplementary Fig. S14d,e) displayed minor impacts on the posterior time estimates of bacteria (Supplementary Fig. S15c).

Next, the use of different genes in dating may lead to different time estimates. Mitochondrial genes tend to evolve faster than their orthologs in bacterial genomes (Martijn et al. 2018; Wang and Luo 2021). In theory, the issue can be accommodated by relaxed molecular clock models, but this is not guaranteed (Drummond et al. 2006; Yang and Rannala 2006). Also, MCMCtree cannot determine which tree it should take. Instead, it takes a fixed tree topology during timetree inference, and so it does not integrate over alternative tree topologies that may be possible due to HGT, unresolved paralogy, or limited phylogenetic signal of individual genes. Nevertheless, if there are contested tree hypotheses that could be tested for a given phylogeny, it is possible to run a Bayesian model selection analysis with both MCMCtree and the R package mcmc3r (dos Reis et al. 2018). To minimize the bias caused by gene selection, we ranked the 19 mitochondrial genes by 1) the relative difference in their estimated evolutionary rates between eukaryotic and bacterial lineages and 2) △LL, an index to measure the species- and gene-tree incongruence (Moody et al. 2022) (see Methods). We selected the 5, 10, and 15 top-ranking (best) genes using each of the above 2 strategies and repeated clock-dating analyses (Fig. 3d). Additionally, we analyzed the full set of 32 mitochondrial genes (*all_genes* in Fig. 3d). Most analyses showed consistent results compared with using the 19-gene data set; the schemes using only the 5 top-ranking genes (schemes *top5*) displayed larger uncertainty in the date estimates of certain nodes (e.g., MBDD [Myxococcota, Bdellovibrionota, Desulfomonadota, and Desulfobacterota]), but this is likely due to their small number of genes.

Lastly, other factors potentially affecting the dating analysis were examined. The largest change in posterior time estimates was observed when the ARs model, which was consistently supported as the preferred model in the Bayesian model selection analysis with mcmc3r (Supplementary Fig. S5 and Supplementary Data S4), was replaced with the poorly supported IR model: the divergence times of most lineages shifted toward the present by ~20% (*IR* in Fig. 3c). The birth-death process with species sampling (Yang and Rannala 1997) is used to specify the prior on those nodes whose age is not constrained by any calibration (i.e., uncalibrated nodes). To generate the joint distribution of all node ages, both this process and the priors used as calibrations will be considered, which may result in a complicated joint prior. Additionally, the impact of changing the parameters of the birth-death process with species sampling was not significant (*bd* in Supplementary Fig. S15b). In addition, analysis with an independently sampled and expanded data set with 285 bacterial genomes (Coleman et al. 2021) showed similar posterior time estimates (*Secondary* in Supplementary Fig. S16), suggesting that the time estimates presented in the main analysis were not dependent on a specific taxon sampling. Note that the overall acceptance rate of the rejection sampling was less than 5%, meaning that more than 95% of the posterior samples were removed in Step 3 of the pRTC framework (see Methods). Because the running time of MCMCtree is roughly proportional to the number of partitions, to save time, all analyses were performed on a single partitioned alignment, but similar patterns were found when 2 partitions were used (Supplementary Fig. S17). In our timetree inference analysis, the calibration densities (i.e., node age constraints we specified, see Supplementary Note S1.2.2) differed from the marginal densities to be used by the dating program (also known as “effective priors”; solid vs. dashed lines in Supplementary Fig. S18 [focal strategy]) due to the overlap between various node age constraints we specified. This truncation issue is common in deep phylogeny dating given that the root age may be constrained with an arbitrary maximum age due to the uncertainty surrounding very ancient events. To alleviate the truncation effect, we assessed the impact of 3 additional sets of calibrations with less overlapping constraints, which resulted in more similar calibrations and marginal densities. The resulting posterior time estimates were very close to those obtained when using the “focal” strategy (Supplementary Fig. S18).

The convergence of all MCMC analyses was checked by comparing the time estimates of 2 independent chains (Supplementary Fig. S19) and by calculating the effective sample size (ESS) to ensure that the ESS of almost all parameters after rejection sampling was above 200 for any single chain in all MCMCtree analyses (Supplementary Data S5).

## DISCUSSION

A major challenge in dating bacterial evolution is the paucity of credible calibrations, particularly the maximum time constraints (Wang and Luo 2021). Previously, this was usually done by assigning a somewhat arbitrary maximum age to the root (LBCA), which greatly varies between studies due to the uncertainty in palaeobiological evidence and phylogenetic relationships around the origin of life (Knoll 2015; Moody et al. 2022). We addressed this problem by leveraging the timing information of eukaryotes based on ancient symbiosis. This provides 2 additional layers of timing information besides bacterial calibrations. Firstly, we linked the evolution of bacteria and eukaryotes by mitochondrial endosymbiosis. Several eukaryotic calibrations incorporate upper bounds to establish a maximum age based on the most recent geological formation that ought to have fossils of the clade of interest. Consequently, these more reliable maximum ages can help inform bacterial evolution if eukaryotic lineages are incorporated into the tree topology. Secondly, based on RTCs informed by host-symbiont association, the timing of eukaryote evolution directly constrains the origin time of symbionts to be no earlier than their eukaryotic host, which is further propagated across the bacterial phylogeny.

The idea of RTC in molecular dating has recently caught much attention (Davín et al. 2018; Magnabosco et al. 2018; Fournier et al. 2021). However, to the best of our knowledge, none of the available RTC applications has considered the uncertainty in the inference of RTC. In other words, previously, the relative time order established by HGT (i.e., gene recipient lineage cannot be older than gene donor lineage) or any other forms of RTC was assumed to be known with certainty, which might be oversimplified due to the uncertainties in identifying these evolutionary events. Our new approach removes this assumption by weighting the different ancestral hosts by their probability established in ASR through a rejection sampling framework. This greatly extends the RTCs based on host-symbiont co-evolution to dating the bacterial tree, which involves many ancient symbioses and large uncertainty in assigning the ancestral host. In addition, prior studies that have applied the idea of host-symbiont association in molecular dating typically used time estimates of the host from other studies (i.e., secondary calibrations) to calibrate symbiont evolution, which may cause wrong time estimates by propagating errors associated with the original time estimates (Schenk 2016). Our strategy co-estimates the origin times of hosts and symbionts based on mitochondrial endosymbiosis (Wang and Luo 2021), avoiding methodological and data differences in comparing posterior time estimates from different studies.

Great care should be taken when interpreting the estimated timeline of the bacterial tree of life and reconstructed evolutionary events taking place anciently. One notable issue is that reconstructing the ancestral lifestyle of bacteria is a very difficult task so there is still much uncertainty associated with the inference (Dharamshi et al. 2020, 2023). Accordingly, although we have spent many efforts minimizing this uncertainty (Fig. 3c, Supplementary Figs. S5 and S10, and Supplementary Note S3.1), the ASR results could still have some impacts on the estimated timetree. Nonetheless, it is remarkable to note the congruence between time estimates obtained using different time constraints, different root positions, independent sets of genomes, different parameters in molecular clock analysis, and, particularly, different settings in ASR which could lead to different ancestral lifestyles, and thus different RTCs. Our time estimate of LBCA is ~0.5 Gyr (billion years) younger than estimated in some studies (Battistuzzi and Hedges 2009; Marin et al. 2017) but is consistent with a recent study using modern phylogenetics software and carefully selected HGT-based time constraints (Fournier et al. 2021). As evident in Fig. 3a, this might be due to the lack of enough maximum time constraints and the use of poorly fit substitution models for inferring deep phylogenies in those early efforts. Moreover, we conducted molecular clock inferences based on a phylogeny of the bacterial tree of life constructed with state-of-the-art phylogenomics methods. Consequently, some groups once thought basal in the bacterial tree, for example, Aquificae (Battistuzzi and Hedges 2009) and CPR (Hug et al. 2016), are suggested as later-split lineages in recent studies (Coleman et al. 2021; Moody et al. 2022) and the present study based on improved phylogenetics methods, leading to different time estimates of corresponding lineages.

In addition, our model assumes the ancestral bacterium took only 1 host or lifestyle at 1 time. While it is straightforward to assume so, it is possible that some symbionts adapt to different lifestyles at different life stages or under different environmental conditions. Further, while bacterial symbionts isolated from animals are typically classified as animal-associated, some might actually be symbionts of the protists that live in the gut or other parts of the animals (Collingro et al. 2020). Additionally, some bacteria found in animal organs like the gut may be transient free-living bacteria, as these kinds of organs are connected to external environments. In sum, misclassification of bacterial lifestyles may indeed occur, which could affect ASR. Nonetheless, note that the internal nodes that served as the RTCs were selected from classical “symbiont clades” based on not only ASR but also a comprehensive literature review (Supplementary Note S3.1). Furthermore, the consistent results obtained when alternative ways of lifestyle classification and parameters in ASR were used suggest that the above caveats likely did not have a large impact on the posterior date estimate in the RTC framework. This is likely because of the many RTCs included such that any change in a few of them is unlikely affected the overall pattern.

The estimated timescale of bacterial evolution allows testing hypotheses involving early life evolution. For example, it is generally believed that the late heavy bombardment likely occurred on early Earth ~4.1–3.8 Ga, during which large asteroids intensely pummeled Earth, melted most parts of its surface, and killed off any forms of life that might have begun to emerge (Abramov and Mojzsis 2009; Boehnke and Harrison 2016). Because the primordial form of life likely had existed before LBCA, our estimates for the age of LBCA at 4.0–3.5 Ga hint that life had survived the cataclysm, consistent with modeling studies showing that much of Earth’s crust and the microbes living therein were unlikely affected by the intense bombardment (Abramov and Mojzsis 2009)., Under the most parsimonious hypothesis that oxygenic cyanobacteria arose in the cyanobacterial stem lineage (Sánchez-Baracaldo and Cardona 2020; Fournier et al. 2021), our analysis placed the appearance of the first oxygenic cyanobacteria between 3.0 Ga (total-group cyanobacteria) and 2.5 Ga (crown-group cyanobacteria) (Fig. 2). This reinforces the previous argument (Hofmann et al. 1999; Allwood et al. 2006) that one cannot link stromatolites found in ancient sediments dated ~3.5 Ga to oxygenic cyanobacteria despite their dominance in stromatolites nowadays. This is supported by recent evidence showing that stromatolites preserved in Archean rocks neither required oxygen-producing cyanobacteria for formation (Schopf 2011) nor were necessarily biogenic (McLoughlin et al. 2008). Additionally, our approach effectively constrained the total group and crown group of oxygenic cyanobacteria (Figs. 2 and 3d). These ages were controversial in previous studies using either cyanobacteria fossils or a combination of both cyanobacteria and plant fossils by a plastid-endosymbiosis strategy (Sánchez-Baracaldo et al. 2014, 2017; Fournier et al. 2021; Zhang et al. 2023).

The new bacterial timetree also makes it possible to infer the mechanisms that drive the origin of enzymes that involve oxygen. Accumulating evidence from isotope analyses suggests the presence of oxygen on Earth back to 3.1 Ga (Crowe et al. 2013; Planavsky et al. 2014; Wang et al. 2018), which might be produced by cyanobacteria as mentioned above, and/or abiotically from hydrogen peroxide (H_2_O_2_) (He et al. 2021), potentially leading to the formation of “oxygen oasis” thus extracellular access to O_2_. A recent study (Jabłońska and Tawfik 2021) tracked the origin of most oxygen-utilizing and -producing enzyme families to the Gracilicutes-Terrabacteria split, which according to our estimate is ~3.6 Ga, predating the above events for ~0.5 Ga (Fig. 2). This suggests that life had access to oxygen earlier than the GOE (Jabłońska and Tawfik 2021), either extra- or intracellularly (e.g., oxygen production through nitric oxide dismutation in Thaumarchaea [Kraft et al. 2022] and the NC10 group [Ettwig et al. 2010]) but more likely the latter.

Our work shows that there is much more information beyond bacterial fossils for calibrating bacterial evolution. The estimated timeline of bacterial evolution may well serve as a foundation for studying the evolution of bacteria, their diversification rate, and their correlation with Earth’s geochemistry. It is hopeful that some of these questions can be answered, and competing hypotheses can be reconciled by future molecular clock studies in combination with modern phylogenetics and comparative genomics methods.

## Supplementary Material

Data available from the Dryad Digital Repository: https://dx.doi.org/10.5061/dryad.1c59zw42s

syae071_suppl_Supplementary_Data_S1-S5

syae071_suppl_Supplementary_Materials

## Data Availability

The scripts to do pRTC and bootstrap-based approach Hessian approximation for MCMCtree are available at https://github.com/evolbeginner/rrtc and https://github.com/evolbeginner/bs_inBV/ respectively. Other scripts and data are available at https://doi.org/10.5061/dryad.1c59zw42s.
